# A prediction model based on the prognostic nutritional index for grade 2 or higher radiation-induced oral mucositis in patients with nasopharyngeal carcinoma

**DOI:** 10.3389/fonc.2026.1951437

**Published:** 2026-09-18

**Authors:** Deyue Jiang, Ruohan Wang, Jin Lu, Huanhuan Yu, Haoxuan Zhang

**Affiliations:** 1 Department of Human Anatomy, Bengbu Medical University, Bengbu, China; 2 Bengbu Hospital of Traditional Chinese Medicine, Bengbu, China

**Keywords:** machine learning, nasopharyngeal carcinoma, prediction model, prognostic nutritional index, radiation-induced oral mucositis

## Abstract

**Introduction:**

To develop a prediction model based on the prognostic nutritional index for grade 2 or higher radiation-induced oral mucositis in patients with nasopharyngeal carcinoma, providing a basis for early identification of patients at high risk.

**Methods:**

We retrospectively included 192 patients with primary nasopharyngeal carcinoma who received chemoradiotherapy at the First Affiliated Hospital of Bengbu Medical University between May 2020 and December 2025. Patients were randomly assigned in a 7:3 ratio to a training set (n = 133) and an internal validation set (n = 59). Baseline characteristics were compared after tests of normality and homogeneity of variance. Candidate variables were selected jointly using LASSO, random forest, and support vector machine recursive feature elimination, followed by univariable and multivariable logistic regression to identify factors associated with grade 2 or higher radiation-induced oral mucositis. A nomogram was constructed, and Firth penalized likelihood regression was used to reduce small-sample bias. Model performance was assessed using receiver operating characteristic analysis and calibration curves, while clinical utility was evaluated by decision curve analysis. Propensity score matching was then used for sensitivity analysis.

**Results:**

The prognostic nutritional index was an independent protective factor of grade 2 or higher radiation-induced oral mucositis. The area under the curve was 0.842 in the training set and 0.725 in the validation set. Patients in the group with high prognostic nutritional index values had a markedly lower risk (OR = 0.067, P < 0.001), and the finding remained consistent in the Firth regression analysis. The association also remained significant after 1:1 propensity score matching (OR = 0.709, P < 0.001; AUC = 0.810).

**Conclusion:**

The prognostic nutritional index is an independent predictor of grade 2 or higher radiation-induced oral mucositis, and the model showed useful predictive performance for this outcome in patients with NPC receiving chemoradiotherapy.

## 1 Introduction

Nasopharyngeal carcinoma (NPC) is an invasive malignant tumor that commonly arises in the pharyngeal recess and the lateral or superior wall of the nasopharynx (1). It has the highest incidence among malignancies of the ear, nose, and throat, and its pathogenesis is complex and influenced by multiple factors (2). NPC has a marked geographic distribution and is particularly prevalent in East and Southeast Asia (3), with China reporting the highest incidence (4). Radiotherapy is a principal curative treatment for NPC, but damage to normal tissues is difficult to avoid and can cause a range of acute toxicities (5).

Radiation-induced oral mucositis (RIOM) is among the most common acute toxicities during radiotherapy for head and neck cancer, with a meta-analysis reporting a prevalence of approximately 94% in this population (6). RIOM may cause ulceration, pain, dysphagia, nutritional deterioration, and treatment interruption, substantially reducing patients’ quality of life. As RIOM progresses, impaired oral intake and worsening nutritional status may further affect patients’ tolerance of anticancer treatment. Current management is largely reactive and includes analgesia, nutritional support, infection control, and other targeted measures. Therefore, early identification of patients at increased risk of RIOM is important for timely preventive and supportive interventions (7, 8).

Pretreatment risk assessment may help identify patients who are more likely to develop clinically significant RIOM before treatment begins. Routine laboratory indicators are readily available and can be repeatedly measured in clinical practice, providing a practical approach for pretreatment risk stratification. The prognostic nutritional index (PNI), calculated from serum albumin and lymphocyte count, reflects both nutritional reserves and immune status (9). Given the association between RIOM and nutritional deterioration, PNI may have potential value as a readily available indicator for pretreatment risk assessment.

In recent years, PNI has received increasing attention as a predictor of adverse outcomes in several cancers (10). Several studies have also examined its association with treatment-related toxicity (11–13). However, evidence regarding the predictive value of pretreatment PNI for grade 2 or higher RIOM in patients with NPC remains limited. Therefore, this study aimed to evaluate the association between pretreatment PNI and grade 2 or higher RIOM in patients with NPC and to develop and internally validate a PNI-based prediction model, thereby providing evidence for early risk stratification and clinical decision-making.

## 2 Materials and methods

### 2.1 Study population

The study included 192 patients with NPC who received chemoradiotherapy at the First Affiliated Hospital of Bengbu Medical University between May 2020 and December 2025.

Inclusion criteria:

(1) first histopathologic diagnosis of NPC; (2) no history of a malignant tumor at another site; (3) received both radiotherapy and chemotherapy during hospitalization; (4) complete and accessible electronic medical records; and (5) complete pretreatment laboratory data and records of adverse-event assessments during radiotherapy in the hospital database.

Exclusion criteria:

(1) severe dysfunction of the heart, liver, kidneys, or other major organs, or another serious underlying disease likely to affect treatment outcomes; (2) missing key laboratory results before radiotherapy, preventing calculation of the study variables; (3) missing core clinical information, preventing RIOM grading; and (4) an uncontrolled severe infection before radiotherapy.

RIOM severity was assessed according to the Radiation Therapy Oncology Group (RTOG) criteria (14). Grade 0 indicated no symptoms. Grade I was defined as mucosal erythema with mild pain that did not require analgesics or interfere with eating. Grade II involved mucosal inflammation and exudation with moderate pain requiring analgesics; patients could eat a semiliquid diet. Grade III was characterized by fibrinous mucositis and severe pain requiring narcotic analgesics, with intake limited to liquids. Grade IV involved ulceration, bleeding, and tissue necrosis, causing severe pain and marked interference with eating. Assessments were performed by physicians who received standardized training. RIOM was graded at least once a week throughout radiotherapy. The highest grade recorded during the full course of radiotherapy was used as the outcome. In the training set, 81 of 133 patients (60.9%) developed grade 2 or higher RIOM, while 52 (39.1%) had RIOM below grade 2. In the validation set, 36 of 59 patients (61.0%) developed grade 2 or higher RIOM, while 23 (39.0%) had RIOM below grade 2. The distribution of grade 2 or higher RIOM was therefore similar in the two datasets.

### 2.2 Data preparation

Baseline clinical data and laboratory results obtained within 7 days before radiotherapy were entered into a standardized Excel worksheet. Variables with more than 15% missing data were excluded. For variables with 15% or less missing data, continuous variables were imputed using the median and categorical variables using the mode. The clinical variables included sex, age, hematologic measures, biochemical measures, and systemic inflammatory markers. Categorical variables were numerically encoded. The outcome and sex were coded as binary variables: grade 2 or higher RIOM = 1 and RIOM below grade 2 = 0; male = 1 and female = 0.

### 2.3 Laboratory measurements

All laboratory measurements were obtained within 7 days before radiotherapy after hospital admission and were analyzed using standardized procedures. Venous blood samples were centrifuged at 3,500 r/min for 10 minutes and used for hematologic and biochemical testing. Complete blood count variables, including white blood cell, lymphocyte, and neutrophil counts, were measured with an automated hematology analyzer. Biochemical measures were obtained from serum collected during the same period in accordance with the procedures of the International Federation of Clinical Chemistry and Laboratory Medicine (IFCC). Systemic inflammatory markers, such as the neutrophil-to-lymphocyte ratio (NLR), were calculated from routine blood counts. All tests were completed within 2 hours of blood collection to maintain data stability and consistency. PNI was used to assess pretreatment nutritional and immune status and was calculated as follows: PNI = serum albumin (g/L) + 5 × absolute lymphocyte count (10^9/L) (15, 16). Because PNI incorporates albumin and lymphocyte count, only PNI was retained in subsequent analysis to avoid duplicated information and collinearity. Peripheral blood lymphocyte percentage (LYM%) is not included in the PNI calculation and reflects immune status from a proportional perspective; it was therefore retained as an independent variable.

### 2.4 Model development

The dataset was randomly divided 7:3 into a training set (n = 133) and a validation set (n = 59). The training set was used to develop the model, and the validation set was used to assess its final predictive performance. Given the limitations of any single variable-selection method (17), three machine learning methods were applied in the training set to identify potential predictors: least absolute shrinkage and selection operator (LASSO), random forest (RF), and support vector machine recursive feature elimination (SVM-RFE). LASSO regression uses an L1 regularization penalty to shrink coefficients and select variables. RF estimates variable importance within an ensemble learning framework. SVM-RFE recursively removes low-weight features to identify an optimal feature subset. Because the methods selected different variables, the union of their results was entered into univariable and multivariable logistic regression analysis to retain potentially informative predictors. Firth penalized likelihood regression was used to reduce estimation bias associated with the small sample and rare events, and a nomogram was constructed.

### 2.5 Model evaluation

Model discrimination was assessed using the area under the receiver operating characteristic curve (ROC AUC), calibration using calibration curves, and clinical utility using decision curve analysis (DCA). Firth penalized likelihood regression was used to reduce rare-event bias. A sensitivity analysis was also performed using 1:1 nearest-neighbor propensity score matching with a caliper of 0.2. After matching, the association between the selected variable and the outcome, as well as model performance, was reassessed.

### 2.6 Statistical analysis

All statistical analyses were performed in R version 4.5.0. All tests were two-sided, and P < 0.05 was considered statistically significant. Continuous variables were assessed for normality and homogeneity of variance. Variables with a normal distribution and equal variances were compared using the independent-samples t test and are presented as mean ± standard deviation ( $\bar{\text{x}}$ ± s). Non-normally distributed variables were compared using the Wilcoxon rank-sum test and are presented as median (P25, P75). Categorical variables were compared using the chi-square test or Fisher’s exact test.

## 3 Results

### 3.1 Patient characteristics

Records from 574 patients with NPC were reviewed. After the inclusion and exclusion criteria were applied, 192 eligible patients were included. All patients received chemoradiotherapy. The patient selection process is shown in **Figures 1**. Patients were divided into a group with RIOM below grade 2 (n = 75) and a group with grade 2 or higher RIOM (n = 117) according to the highest grade recorded during treatment. The overall incidence of grade 2 or higher RIOM was 60.94%. The cohort included 145 men (75.52%) and 47 women (24.48%), with no difference in sex distribution between the groups (P = 1.000). The mean age was 53.43 ± 13.32 years; the median age was 53 years, with a range of 7 to 83 years. Baseline demographic and clinical characteristics are shown in **Table 1**. Age, NEUT%, and several other variables differed significantly between the groups (P < 0.05), while the remaining variables did not. Baseline imbalances were adjusted in subsequent analysis.

**Figure 1:**
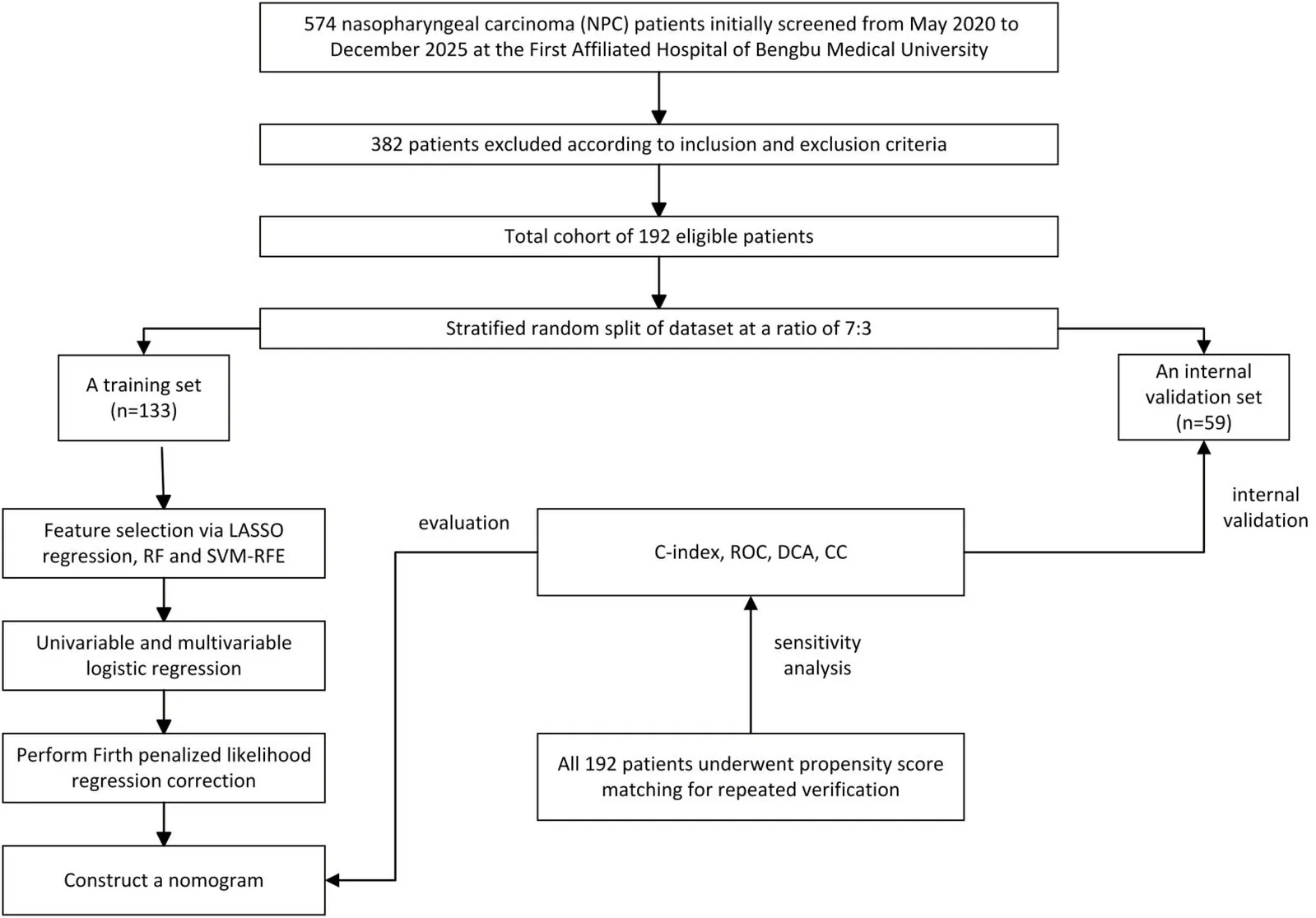
Study flowchart.

**Table 1:** Baseline characteristics of patients with NPC according to RIOM status.

Variable	Overall (N = 192)	Group		P value
		Grade < 2 (n = 75)	Grade ≥ 2 (n = 117)	
Sex				
0	47	18	29	1.000
1	145	57	88
Age	53.00 (45.00, 63.25)	50.00 (43.00, 60.50)	54.00 (47.00, 64.00)	0.035
WBC	5.82 (4.98, 7.27)	6.06 (5.15, 7.11)	5.59 (4.78, 7.37)	0.193
NEUT	3.71 (2.83, 5.00)	3.73 (2.83, 4.86)	3.70 (2.83, 5.04)	0.920
EOS	0.11 (0.07, 0.20)	0.14 (0.08, 0.22)	0.11 (0.06, 0.18)	0.151
BASO	0.02 (0.01, 0.03)	0.02 (0.01, 0.03)	0.02 (0.01, 0.03)	0.948
MONO	0.40 (0.32, 0.51)	0.41 (0.32, 0.53)	0.39 (0.32, 0.51)	0.841
NEUT%	63.58 ± 10.09	60.66 ± 10.21	65.44 ± 9.59	0.001
LYM%	26.28 ± 9.18	29.23 ± 8.98	24.38 ± 8.84	<0.001
EOS%	2.10 (1.10, 3.20)	2.30 (1.20, 3.30)	2.00 (1.10, 3.10)	0.410
BASO%	0.30 (0.20, 0.50)	0.30 (0.20, 0.50)	0.30 (0.20, 0.50)	0.499
MONO%	6.70 (5.78, 7.75)	6.60 (5.65, 7.80)	6.90 (6.00, 7.70)	0.332
RBC	4.61 (4.26, 4.88)	4.68 (4.33, 5.02)	4.57 (4.24, 4.79)	0.035
HGB	140.00 (130.00, 150.00)	142.00 (133.00, 155.00)	139.00 (130.00, 147.00)	0.056
HCT	0.42 (0.39, 0.45)	0.43 (0.40, 0.46)	0.42 (0.39, 0.44)	0.032
MCV	91.45 (88.97, 95.10)	90.30 (88.40, 95.10)	91.70 (89.40, 94.90)	0.185
MCH	30.70 (29.80, 31.42)	30.70 (29.70, 31.40)	30.80 (29.90, 31.40)	0.578
MCHC	334.62 ± 9.56	335.01 ± 9.23	334.38 ± 9.80	0.653
RDW_CV	12.70 (12.20, 13.40)	12.80 (12.35, 13.40)	12.70 (12.20, 13.30)	0.229
RDW_SD	41.45 (40.00, 43.42)	42.00 (40.15, 43.30)	41.40 (39.90, 43.70)	0.459
PLT	243.50 (201.00, 277.50)	245.00 (208.50, 267.50)	242.00 (199.00, 279.00)	0.727
MPV	10.10 (9.57, 10.90)	10.10 (9.65, 10.80)	10.10 (9.40, 10.90)	0.660
PCT	0.25 (0.22, 0.28)	0.25 (0.22, 0.27)	0.25 (0.21, 0.28)	0.961
PDW	15.90 (13.07, 16.20)	15.80 (12.05, 16.20)	15.90 (13.60, 16.20)	0.096
P_LCR	27.00 (21.77, 32.42)	27.00 (21.95, 31.05)	27.00 (21.40, 32.60)	0.985
RET	0.05 (0.04, 0.06)	0.05 (0.04, 0.06)	0.05 (0.04, 0.06)	0.551
RETP	1.05 (0.81, 1.32)	1.05 (0.85, 1.32)	1.05 (0.81, 1.32)	0.916
ALT	16.00 (13.00, 24.00)	17.00 (13.00, 27.00)	16.00 (13.00, 20.00)	0.370
AST	20.00 (17.00, 23.00)	20.00 (17.00, 24.00)	20.00 (16.00, 22.00)	0.424
ALP	72.00 (58.75, 87.00)	69.00 (58.50, 85.50)	72.00 (59.00, 87.00)	0.409
GGT	23.00 (16.00, 33.00)	22.00 (16.00, 34.00)	23.00 (16.00, 33.00)	0.871
ChE	7775.00 (6901.50, 8883.75)	8086.00 (7125.00, 9434.50)	7513.00 (6697.00, 8524.00)	0.046
TBIL	8.50 (6.28, 11.60)	9.30 (7.00, 12.20)	7.80 (6.00, 10.20)	0.021
DBIL	3.00 (2.38, 4.20)	3.30 (2.35, 4.45)	2.90 (2.40, 3.70)	0.160
IBIL	5.35 (3.80, 7.33)	6.10 (4.40, 8.35)	5.20 (3.40, 6.70)	0.006
TBA	3.50 (2.30, 5.45)	3.20 (2.05, 5.75)	3.50 (2.50, 4.90)	0.356
PA	240.00 (210.00, 280.00)	250.00 (218.00, 285.00)	240.00 (200.00, 270.00)	0.059
TP	70.00 (66.17, 73.83)	71.20 (68.95, 74.85)	68.90 (65.50, 73.10)	<0.001
GLOB	29.15 (26.38, 32.50)	29.40 (26.45, 31.90)	29.10 (26.10, 32.50)	0.709
A/G	1.40 (1.20, 1.50)	1.40 (1.30, 1.60)	1.40 (1.20, 1.50)	0.019
RBP	41.05 (30.23, 50.00)	37.50 (28.55, 49.50)	41.05 (32.00, 50.00)	0.470
GLU	4.91 (4.46, 5.38)	4.82 (4.39, 5.37)	4.93 (4.60, 5.37)	0.439
UA	275.50 (236.50, 324.25)	295.00 (244.50, 338.50)	269.00 (234.00, 301.00)	0.029
Cr	66.00 (59.00, 73.00)	67.00 (60.00, 74.50)	66.00 (58.00, 72.00)	0.352
BUN	4.80 (4.07, 5.81)	4.57 (4.00, 5.95)	4.90 (4.22, 5.80)	0.310
K	3.80 (3.60, 4.05)	3.71 (3.58, 3.94)	3.81 (3.67, 4.10)	0.082
Na	141.00 (140.00, 143.00)	141.00 (140.00, 143.00)	141.00 (140.00, 143.00)	0.923
CL	102.00 (101.00, 104.00)	103.00 (101.00, 104.00)	102.00 (101.00, 104.00)	0.331
Ca	2.24 (2.16, 2.30)	2.27 (2.20, 2.35)	2.21 (2.14, 2.28)	<0.001
iCa	1.18 (1.13, 1.23)	1.20 (1.12, 1.26)	1.17 (1.13, 1.21)	0.038
P	1.07 (0.93, 1.21)	1.11 (0.95, 1.21)	1.07 (0.93, 1.21)	0.592
HCO3	25.00 (23.20, 27.12)	24.30 (22.90, 26.75)	25.20 (23.90, 27.20)	0.052
AG	14.15 (11.88, 15.80)	14.10 (11.75, 15.80)	14.15 (12.20, 15.80)	0.952
OP	281.90 (278.60, 284.85)	281.90 (279.00, 283.90)	281.90 (278.60, 285.70)	0.366
TC	4.09 (3.64, 4.63)	4.34 (3.60, 4.95)	4.01 (3.66, 4.53)	0.096
TG	1.23 (0.93, 1.61)	1.23 (0.91, 1.60)	1.24 (0.93, 1.64)	0.798
HDL	1.05 (0.89, 1.27)	1.05 (0.89, 1.26)	1.05 (0.89, 1.26)	0.975
LDL	2.64 (2.19, 3.08)	2.66 (2.18, 3.23)	2.64 (2.24, 2.88)	0.254
Apoa	1.14 (1.00, 1.23)	1.14 (0.99, 1.26)	1.14 (1.00, 1.22)	0.666
Apob	0.80 (0.68, 0.88)	0.80 (0.69, 0.89)	0.80 (0.68, 0.85)	0.726
LPa	175.00 (94.00, 268.25)	175.00 (83.00, 276.00)	175.00 (101.00, 267.00)	0.962
Cys-c	0.90 (0.85, 1.00)	0.90 (0.80, 0.98)	0.90 (0.88, 1.01)	0.326
CRP	2.50 (1.09, 6.54)	2.34 (0.79, 5.73)	2.50 (1.30, 6.50)	0.163
SOD	175.00 (156.75, 181.00)	175.00 (161.50, 186.50)	175.00 (153.00, 177.00)	0.041
PNI	48.48 (45.70, 50.20)	52.25 (48.00, 55.20)	47.30 (44.95, 48.90)	<0.001

Continuous variables are presented as mean ± standard deviation when normally distributed and as median (P25, P75) otherwise. Categorical variables are presented as counts. P < 0.05 was considered statistically significant.

WBC, white blood cell count; NEUT, neutrophil count; EOS, eosinophil count; BASO, basophil count; MONO, monocyte count; NEUT%, neutrophil percentage; LYM%, lymphocyte percentage; EOS%, eosinophil percentage; BASO%, basophil percentage; MONO%, monocyte percentage; RBC, red blood cell count; HGB, hemoglobin; HCT, hematocrit; MCV, mean corpuscular volume; MCH, mean corpuscular hemoglobin; MCHC, mean corpuscular hemoglobin concentration; RDW_CV, coefficient of variation of red cell distribution width; RDW_SD, standard deviation of red cell distribution width; PLT, platelet count; MPV, mean platelet volume; PCT, plateletcrit; PDW, platelet distribution width; P-LCR, platelet large cell ratio; RET, reticulocyte count; RETP, reticulocyte percentage; ALT, alanine aminotransferase; AST, aspartate aminotransferase; ALP, alkaline phosphatase; GGT, gamma-glutamyl transferase; ChE, cholinesterase; TBIL, total bilirubin; DBIL, direct bilirubin; IBIL, indirect bilirubin; TBA, total bile acids; PA, prealbumin; TP, total protein; GLOB, globulin; A/G, albumin-to-globulin ratio; RBP, retinol-binding protein; GLU, glucose; UA, uric acid; Cr, creatinine; BUN, blood urea nitrogen; K, potassium; Na, sodium; CL, chloride; Ca, total calcium; iCa, ionized calcium; P, inorganic phosphorus; HCO3, bicarbonate; AG, anion gap; OP, osmolality; TC, total cholesterol; TG, triglycerides; HDL, high-density lipoprotein cholesterol; LDL, low-density lipoprotein cholesterol; ApoA, apolipoprotein A; ApoB, apolipoprotein B; LPa, lipoprotein(a); Cys-C, cystatin C; CRP, C-reactive protein; SOD, superoxide dismutase; PNI, prognostic nutritional index.

### 3.2 Comparison of baseline characteristics

After random allocation, SOD, GLU, MPVand P_LCR differed significantly between the training and validation sets (P < 0.05). No significant differences were observed for the other baseline variables (all P > 0.05), indicating that the datasets were broadly comparable. To address potential confounding caused by these imbalances, a sensitivity analysis was subsequently conducted using 1:1 nearest-neighbor propensity score matching (PSM). Associations and predictive performance were reassessed after matching (**Table 2**).

**Table 2:** Comparison of baseline characteristics between the training and internal validation sets.

Variable	Overall (N = 192)	Group		P value
		Training set (n = 133)	Validation set (n = 59)	
Sex				0.700
0	47	31	16	
1	145	102	43	
Age	53.00 (45.00, 63.25)	52.00 (46.00, 62.00)	56.00 (45.00, 64.00)	0.838
WBC	5.82 (4.98, 7.27)	5.92 (4.98, 7.22)	5.59 (4.98, 7.30)	0.650
NEUT	3.71 (2.83, 5.00)	3.73 (2.84, 5.01)	3.66 (2.71, 4.98)	0.730
EOS	0.11 (0.07, 0.20)	0.11 (0.05, 0.20)	0.12 (0.08, 0.19)	0.240
BASO	0.02 (0.01, 0.03)	0.02 (0.01, 0.03)	0.02 (0.01, 0.03)	0.151
MONO	0.40 (0.32, 0.51)	0.40 (0.32, 0.51)	0.38 (0.32, 0.52)	0.838
NEUT%	63.58 ± 10.09	63.88 ± 10.20	62.90 ± 9.88	0.536
LYM%	26.28 ± 9.18	25.96 ± 9.09	26.99 ± 9.42	0.474
EOS%	2.10 (1.10, 3.20)	1.90 (0.90, 3.20)	2.40 (1.40, 3.25)	0.237
BASO%	0.30 (0.20, 0.50)	0.30 (0.20, 0.40)	0.30 (0.20, 0.55)	0.178
MONO%	6.70 (5.78, 7.75)	6.70 (5.80, 7.90)	6.70 (5.70, 7.60)	0.789
RBC	4.61 (4.26, 4.88)	4.63 (4.31, 4.90)	4.55 (4.12, 4.82)	0.206
HGB	140.00 (130.00, 150.00)	141.00 (132.00, 151.00)	139.00 (125.00, 148.50)	0.414
HCT	0.42 (0.39, 0.45)	0.42 (0.40, 0.45)	0.42 (0.38, 0.45)	0.616
MCV	91.45 (88.97, 95.10)	91.00 (88.70, 94.50)	91.50 (89.90, 95.20)	0.240
MCH	30.70 (29.80, 31.42)	30.70 (29.80, 31.40)	30.80 (29.95, 31.45)	0.668
MCHC	334.00 (328.00, 340.25)	334.00 (329.00, 342.00)	334.00 (327.50, 338.00)	0.348
RDW_CV	12.70 (12.20, 13.40)	12.70 (12.20, 13.30)	12.80 (12.35, 13.40)	0.176
RDW_SD	41.45 (40.00, 43.42)	41.10 (39.90, 43.30)	42.30 (40.65, 43.75)	0.051
PLT	243.50 (201.00, 277.50)	245.00 (205.00, 279.00)	232.00 (198.00, 262.50)	0.180
MPV	10.10 (9.57, 10.90)	10.10 (9.50, 10.70)	10.50 (9.65, 11.30)	0.020
PCT	0.25 (0.22, 0.28)	0.25 (0.21, 0.28)	0.24 (0.22, 0.28)	0.788
PDW	15.90 (13.07, 16.20)	15.90 (12.80, 16.20)	15.90 (13.40, 16.25)	0.422
P_LCR	27.00 (21.77, 32.42)	26.70 (21.40, 30.90)	29.60 (23.30, 35.80)	0.007
RET	0.05 (0.04, 0.06)	0.05 (0.04, 0.06)	0.05 (0.04, 0.06)	0.960
RETP	1.05 (0.81, 1.32)	1.05 (0.81, 1.32)	1.05 (0.81, 1.32)	0.888
ALT	16.00 (13.00, 24.00)	16.00 (13.00, 22.00)	16.00 (13.00, 24.50)	0.906
AST	20.00 (17.00, 23.00)	20.00 (16.00, 23.00)	20.00 (18.00, 22.00)	0.562
ALP	72.00 (58.75, 87.00)	72.00 (60.00, 87.00)	69.00 (55.00, 85.50)	0.173
GGT	23.00 (16.00, 33.00)	23.00 (16.00, 33.00)	23.00 (16.00, 32.00)	0.783
ChE	7775.00 (6901.50, 8883.75)	7814.00 (7041.00, 9026.00)	7639.00 (6502.50, 8530.50)	0.166
TBIL	8.50 (6.28, 11.60)	8.60 (6.30, 11.60)	8.10 (6.15, 11.35)	0.790
DBIL	3.00 (2.38, 4.20)	3.00 (2.40, 4.20)	3.00 (1.90, 4.20)	0.482
IBIL	5.35 (3.80, 7.33)	5.40 (3.80, 7.20)	5.20 (3.90, 7.35)	0.985
TBA	3.50 (2.30, 5.45)	3.40 (2.10, 5.80)	3.90 (3.10, 5.15)	0.176
PA	240.00 (210.00, 280.00)	240.00 (216.00, 276.00)	240.00 (200.00, 286.00)	0.628
TP	70.00 (66.17, 73.83)	70.30 (66.70, 74.20)	69.10 (65.85, 71.95)	0.099
GLOB	29.15 (26.38, 32.50)	29.40 (26.60, 32.50)	29.00 (26.25, 32.25)	0.465
A/G	1.40 (1.20, 1.50)	1.40 (1.20, 1.50)	1.40 (1.20, 1.60)	0.573
RBP	41.05 (30.23, 50.00)	41.05 (31.60, 51.00)	36.00 (28.50, 46.50)	0.159
GLU	4.91 (4.46, 5.38)	4.95 (4.60, 5.45)	4.78 (4.31, 5.01)	0.002
UA	275.50 (236.50, 324.25)	273.00 (230.00, 317.00)	280.00 (245.50, 337.50)	0.215
Cr	66.00 (59.00, 73.00)	66.00 (59.00, 72.00)	67.00 (59.50, 74.00)	0.444
BUN	4.80 (4.07, 5.81)	4.80 (4.00, 5.80)	4.90 (4.20, 5.87)	0.574
K	3.80 (3.60, 4.05)	3.80 (3.60, 4.00)	3.81 (3.61, 4.10)	0.330
Na	141.00 (140.00, 143.00)	141.00 (140.00, 143.00)	142.00 (140.00, 143.00)	0.670
CL	102.00 (101.00, 104.00)	102.00 (101.00, 104.00)	103.00 (101.00, 104.80)	0.340
Ca	2.24 (2.16, 2.30)	2.23 (2.17, 2.29)	2.24 (2.15, 2.30)	0.945
iCa	1.18 (1.13, 1.23)	1.19 (1.14, 1.23)	1.17 (1.12, 1.22)	0.411
P	1.07 (0.93, 1.21)	1.07 (0.95, 1.21)	1.07 (0.92, 1.17)	0.588
HCO3	25.00 (23.20, 27.12)	25.00 (23.30, 27.20)	24.70 (23.00, 26.85)	0.398
AG	13.85 ± 3.39	13.88 ± 3.44	13.78 ± 3.31	0.855
OP	281.90 (278.60, 284.85)	281.90 (278.60, 284.80)	282.30 (278.75, 285.10)	0.810
TC	4.18 ± 0.83	4.19 ± 0.79	4.15 ± 0.92	0.764
TG	1.23 (0.93, 1.61)	1.24 (0.89, 1.61)	1.20 (0.94, 1.56)	0.854
HDL	1.05 (0.89, 1.27)	1.04 (0.89, 1.25)	1.05 (0.88, 1.27)	0.974
LDL	2.64 (2.19, 3.08)	2.64 (2.30, 3.08)	2.52 (2.09, 3.07)	0.235
Apoa	1.14 (1.00, 1.23)	1.14 (1.00, 1.23)	1.14 (1.00, 1.23)	0.755
Apob	0.80 (0.68, 0.88)	0.80 (0.72, 0.88)	0.80 (0.66, 0.83)	0.186
LPa	175.00 (94.00, 268.25)	175.00 (90.00, 248.00)	175.00 (111.50, 284.50)	0.396
Cys-c	0.90 (0.85, 1.00)	0.90 (0.89, 0.98)	0.90 (0.84, 1.10)	0.566
CRP	2.50 (1.09, 6.54)	2.50 (0.90, 5.10)	2.50 (1.23, 8.10)	0.506
SOD	175.00 (156.75, 181.00)	175.00 (162.00, 183.00)	174.00 (149.50, 176.00)	0.024
PNI	48.48 (45.70, 50.20)	48.50 (46.00, 50.35)	48.25 (45.17, 50.15)	0.451

Continuous variables are presented as mean ± standard deviation when normally distributed and as median (P25, P75) otherwise. Categorical variables are presented as counts. P < 0.05 was considered statistically significant.

### 3.3 Variable selection

LASSO selected 5 variables, RF identified 20 variables of importance, and SVM-RFE selected 1 core variable. After duplicate variables were removed, 22 unique variables remained (**Figures 2A–H**).

**Figure 2:**
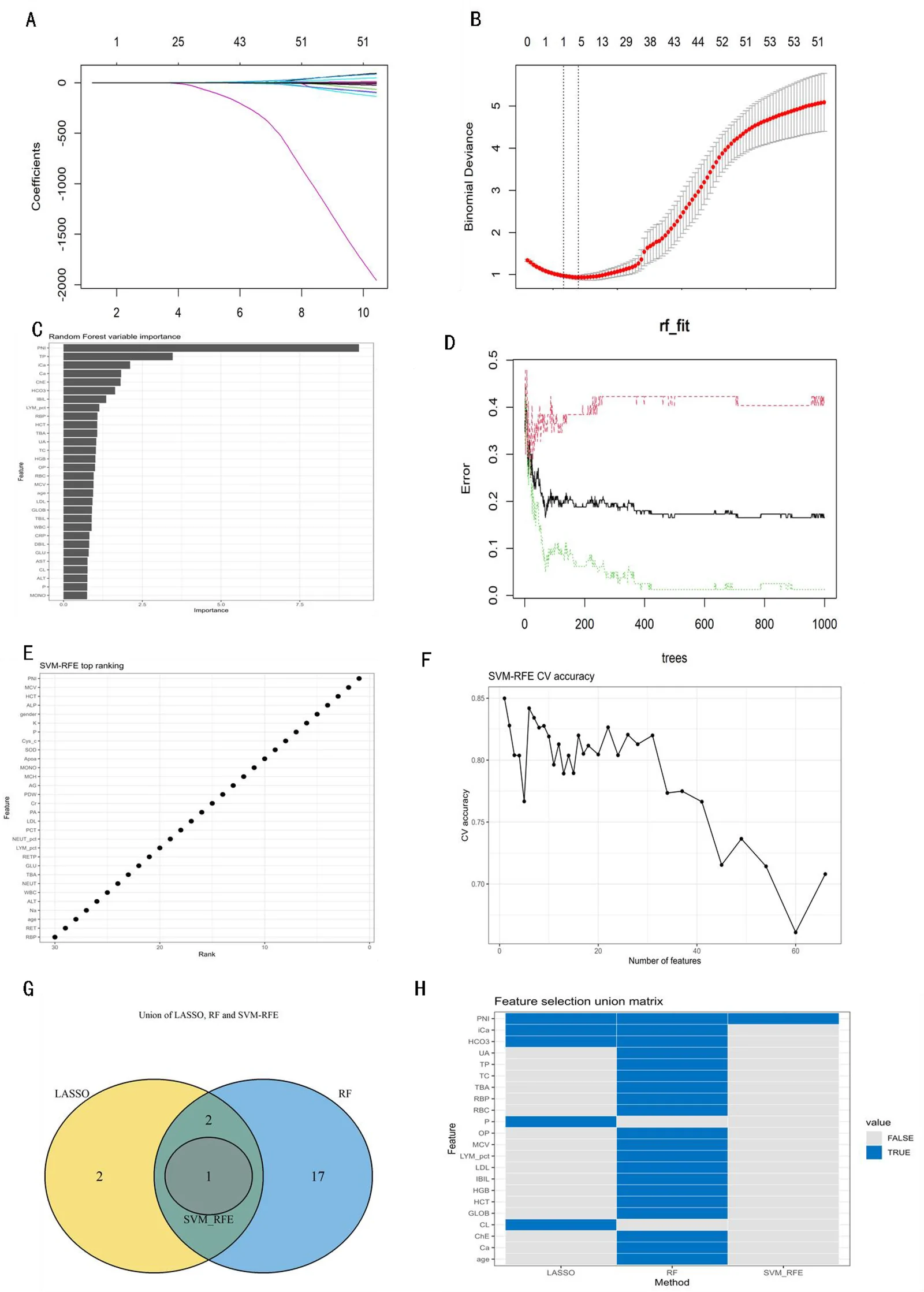
Feature selection using multiple machine learning methods. **(A, B)** feature selection by LASSO regression; **(C, D)** variable selection by random forest; **(E, F)** feature selection by SVM-RFE; **(G)** Venn diagram showing the union of variables selected by the three methods; H, matrix heatmap of variables selected by the three methods.

### 3.4 Logistic regression analysis

Univariable logistic regression was performed for the 22 variables selected by the three machine learning methods. Seven variables were significantly associated with the outcome (P < 0.05; **Table 3**). The remaining variables were not significant and were not entered into the multivariable analysis.

**Table 3:** Univariable logistic regression analysis.

Variable	β	SE	Wald χ²	P value	OR	95% CI
CL	-0.12	0.068	3.076	0.079	0.887	0.776-1.014
iCa	-2.908	2.163	1.806	0.179	0.055	0.001-3.791
P	-0.299	0.347	0.742	0.389	0.741	0.375-1.465
HCO3	0.147	0.065	5.144	0.023	1.158	1.020-1.315
PNI	-0.524	0.099	28.309	<0.001	0.592	0.488-0.718
TP	-0.121	0.035	12.191	<0.001	0.886	0.828-0.948
Ca	-4.993	1.76	8.046	0.005	0.007	0.000-0.214
ChE	0	0	6.991	0.008	1	0.999-1.000
IBIL	-0.105	0.055	3.63	0.057	0.9	0.808-1.003
LYM%	-0.052	0.021	6.042	0.014	0.95	0.911-0.990
RBP	0.006	0.012	0.212	0.645	1.006	0.982-1.030
HCT	-8.239	4.682	3.097	0.078	0	0.000-2.551
TBA	0.063	0.053	1.392	0.238	1.065	0.959-1.181
UA	-0.004	0.003	3.035	0.082	0.996	0.991-1.001
TC	-0.566	0.239	5.611	0.018	0.568	0.355-0.907
HGB	-0.017	0.012	1.844	0.175	0.984	0.960-1.007
OP	0.032	0.027	1.411	0.235	1.033	0.979-1.090
RBC	-0.866	0.441	3.85	0.05	0.421	0.177-0.999
MCV	0.032	0.035	0.821	0.365	1.032	0.964-1.106
age	0.01	0.014	0.528	0.467	1.01	0.983-1.038
LDL	-0.405	0.265	2.341	0.126	0.667	0.397-1.121
GLOB	-0.02	0.034	0.351	0.554	0.98	0.917-1.048

All seven variables that were significant in the univariable analysis were entered into a multivariable logistic regression model. Only PNI remained significant (OR = 0.586, 95% CI 0.463 to 0.741, P < 0.001), indicating that PNI was independently associated with RIOM. None of the other variables reached statistical significance (P > 0.05), suggesting that they had no independent predictive value (**Table 4**).

**Table 4:** Multivariable logistic regression analysis.

Variable	β	SE	Wald χ²	P value	OR	95% CI
PNI	-0.534	0.12	19.854	<0.001	0.586	0.463–0.741
TP	-0.035	0.051	0.452	0.502	0.966	0.873–1.068
Ca	0.993	2.665	0.139	0.71	2.698	0.015–500.943
ChE	0	0	0.275	0.6	1	1.000–1.000
LYM%	0.005	0.031	0.023	0.879	1.005	0.946–1.067
TC	-0.109	0.326	0.111	0.739	0.897	0.473–1.700
HCO3	0.134	0.084	2.563	0.109	1.144	0.970–1.348

To present the odds ratios visually, a forest plot was drawn for the multivariable logistic regression model (**Figure 3**). Odds ratios are shown on a logarithmic scale. The vertical axis lists the candidate variables, points represent OR estimates, horizontal lines represent 95% confidence intervals, and the vertical reference line marks OR = 1. Only the 95% confidence interval for PNI did not cross 1. Its OR was 0.586 (95% CI 0.463 to 0.741, P < 0.001), indicating that PNI remained independently associated with grade 2 or higher RIOM after adjustment for the other variables and acted as a protective factor. The risk of grade 2 or higher RIOM decreased as PNI increased. The confidence intervals for the other variables crossed 1, consistent with the multivariable regression results.

**Figure 3:**
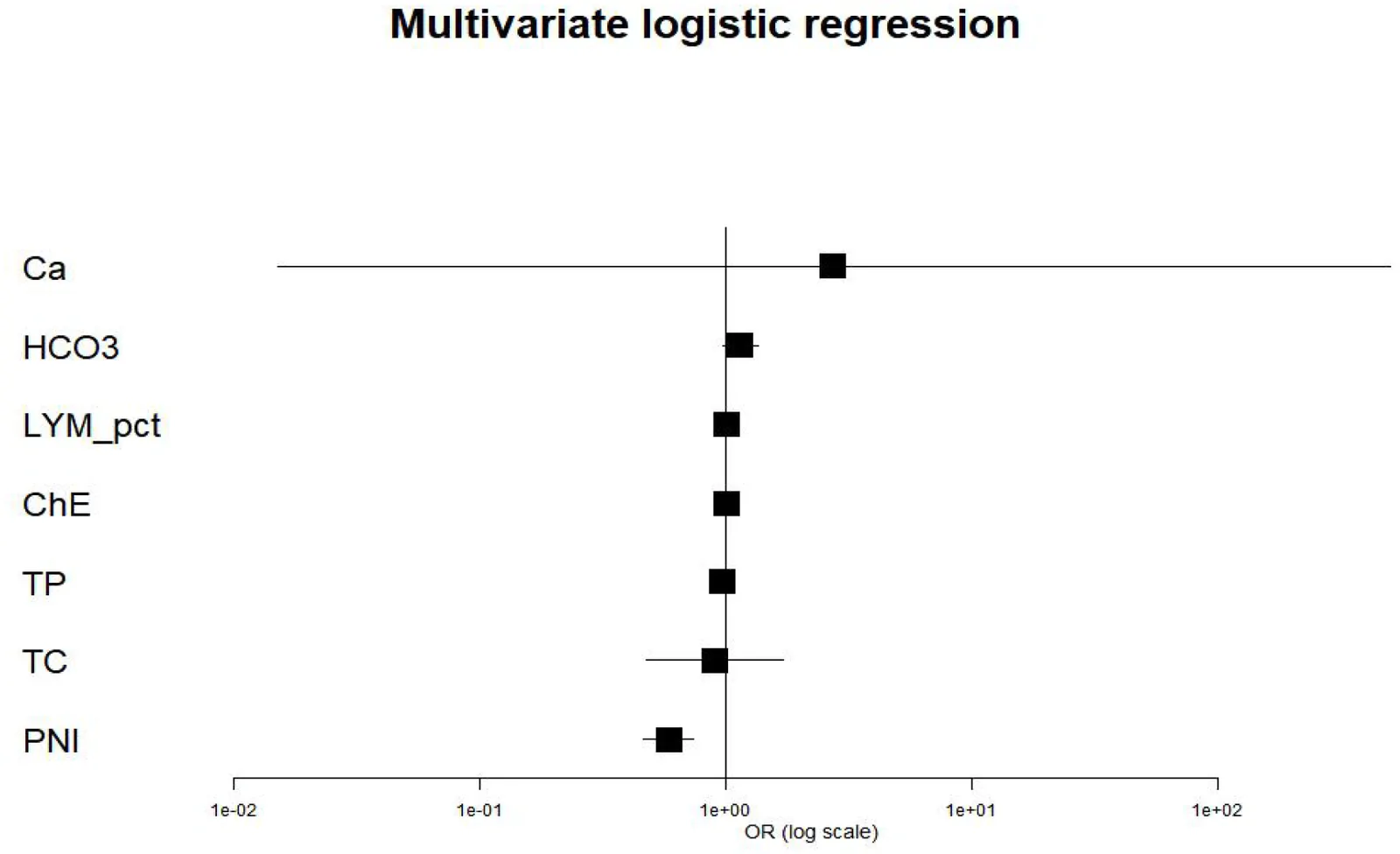
Forest plot for multivariable logistic regression.

### 3.5 Bias-corrected analysis using firth penalized likelihood regression

Multivariable logistic regression identified one independent predictor of grade 2 or higher RIOM in patients with NPC treated with chemoradiotherapy (P < 0.05). To reduce parameter bias in conventional maximum likelihood estimation with a small sample, this predictor was categorized and analyzed using Firth penalized likelihood regression, with the low PNI group as the reference. PNI in the training set was divided into tertiles. The cutoffs were 47.05 and 50.05, defining a low PNI group (≤47.05), an intermediate PNI group (>47.05 to ≤50.05), and a high PNI group (>50.05). As shown in **Table 5**, the risk of severe RIOM decreased with increasing PNI, and the protective association was strongest in the high PNI group.

**Table 5:** Firth penalized likelihood regression analysis of factors associated with RIOM.

Variable	Regression coefficient (β)	Standard error (SE)	Odds ratio (OR)	Lower 95% CI	Upper 95% CI	P value
PNI (low PNI group, reference)	—	—	1	—	—	—
PNI (intermediate vs low PNI)	-0.68	0.52	0.506	0.176	1.379	0.185
PNI (high vs low PNI)	-2.705	0.525	0.067	0.022	0.178	<0.001

### 3.6 Nomogram development and model evaluation

The nomogram based on PNI categories is shown in **Figure 4A**. The AUC was 0.842 in the training set and 0.725 in the validation set, indicating useful discrimination. The calibration curves showed reasonable agreement between predicted and observed probabilities in both datasets. DCA showed that the model provided greater net benefit than the treat-all and treat-none strategies across a broad range of threshold probabilities (**Figures 4A–D**).

**Figure 4:**
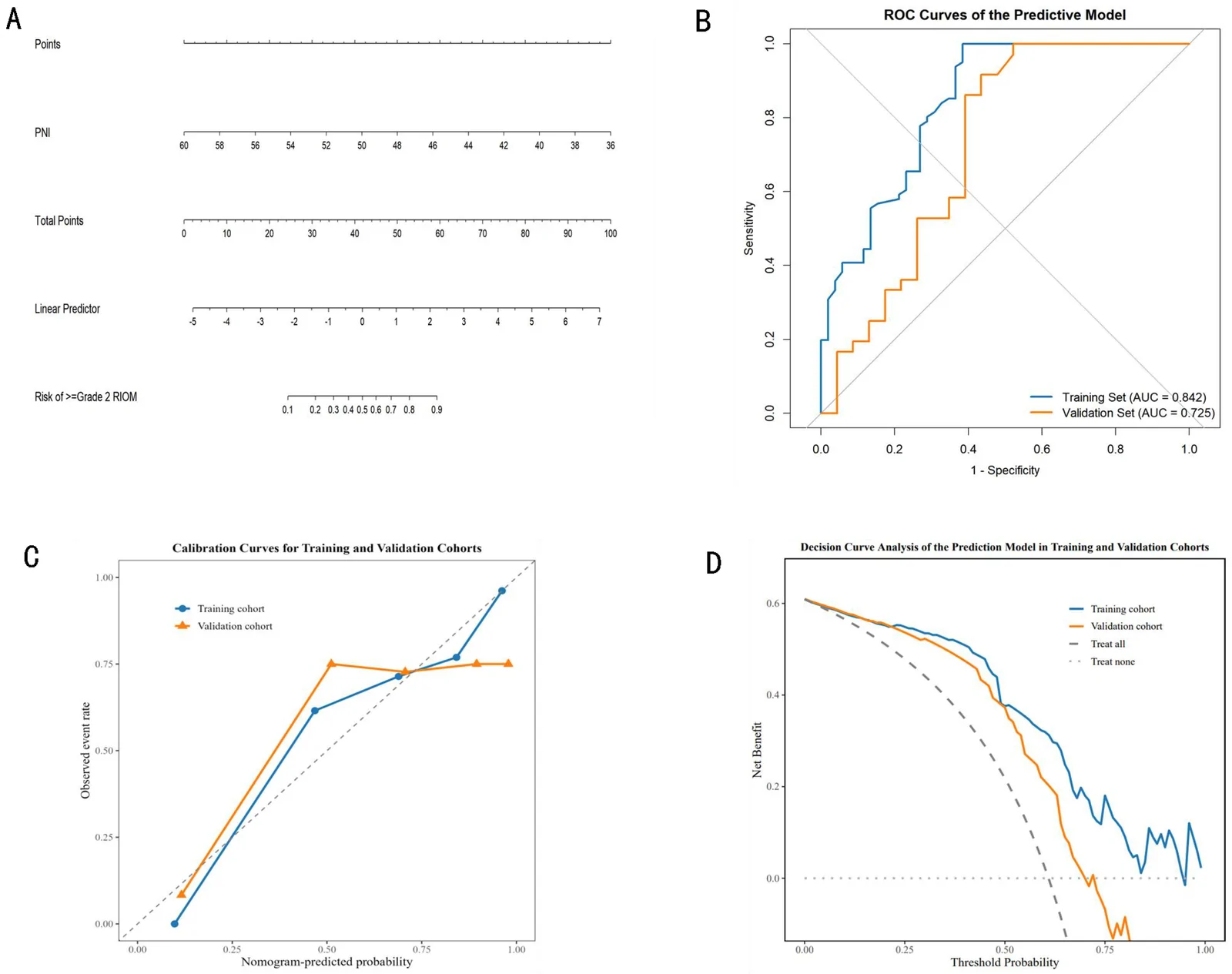
Development and performance evaluation of the PNI-based prediction model. **(A)** Nomogram based on PNI for predicting the risk of grade 2 or higher radiation-induced oral mucositis. **(B)** Receiver operating characteristic curves of the model in the training and validation sets, with AUC values of 0.842 and 0.725, respectively. **(C)** Calibration curves comparing the nomogram-predicted probabilities with the observed event rates in the training and validation sets. **(D)** Decision curve analysis showing the net benefit of the model across different threshold probabilities in the training and validation sets. PNI, prognostic nutritional index; AUC, area under the curve.

### 3.7 Sensitivity analysis

Baseline confounding was addressed using 1:1 PSM. A total of 150 patients with NPC were included after matching, and the baseline characteristics of the two groups were balanced (**Table 6**). In the matched cohort, PNI remained an independent protective factor for grade 2 or higher RIOM (OR = 0.709, 95% CI 0.627 to 0.800, P < 0.001). The AUC in the matched cohort was 0.810 (**Figure 5**), close to that of the original cohort, providing further support for the association.

**Figure 5:**
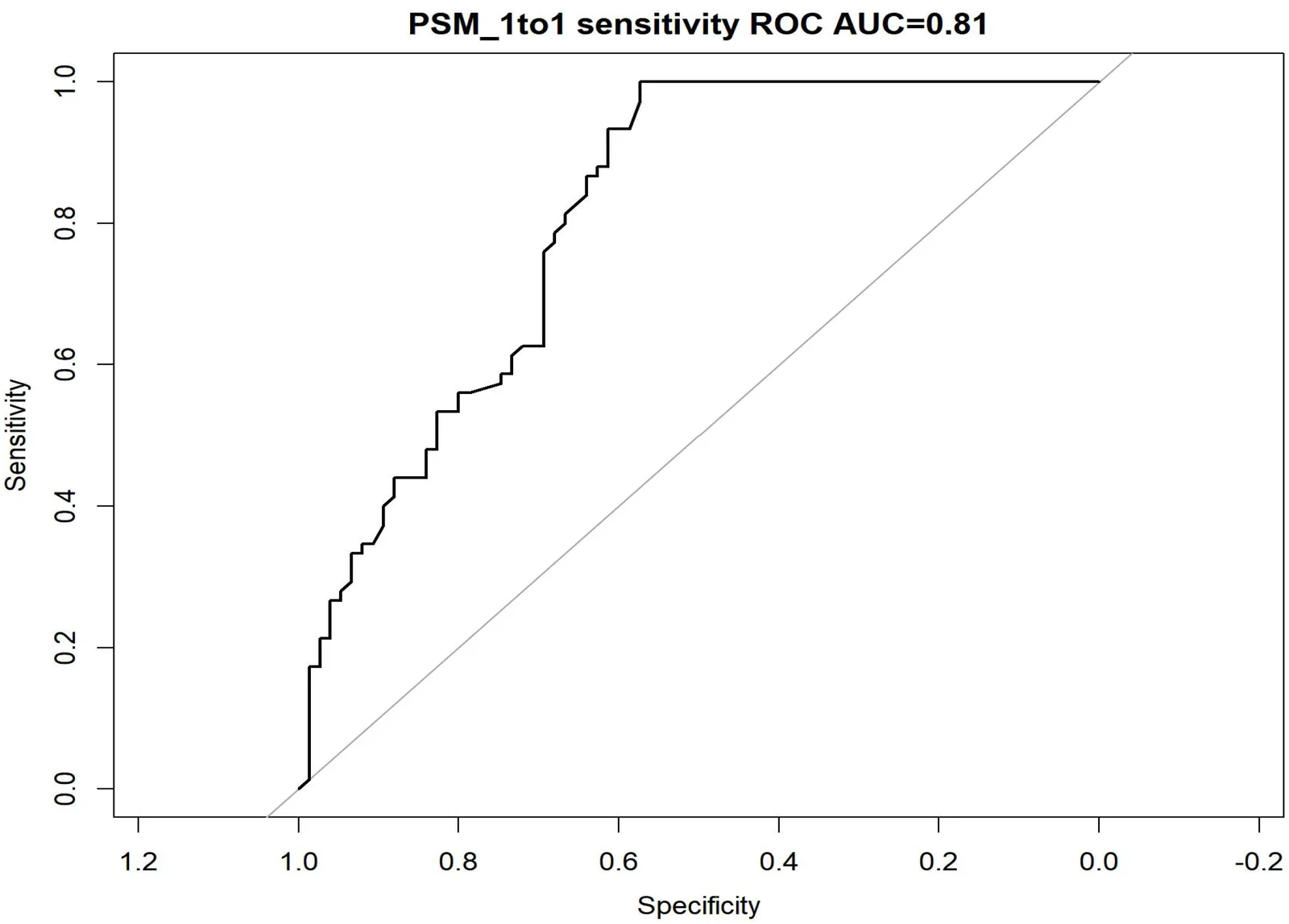
ROC curve of the prediction model developed in the 1:1 propensity score-matched cohort.

**Table 6:** Comparison of baseline clinical characteristics after 1:1 propensity score matching.

Variable	Overall	Control group	Model group	P value
Age	53.44 ± 12.45	52.76 ± 12.52	54.13 ± 12.44	0.515
WBC	5.88 (5.02, 7.33)	6.13 (5.26, 7.11)	5.80 (4.77, 7.43)	0.268
NEUT	3.75 (2.79, 5.00)	3.76 (2.85, 4.86)	3.75 (2.68, 5.07)	0.92
EOS	0.11 (0.06, 0.19)	0.14 (0.08, 0.23)	0.10 (0.06, 0.15)	0.036
BASO	0.02 (0.01, 0.03)	0.02 (0.01, 0.03)	0.02 (0.01, 0.03)	0.948
MONO	0.40 (0.32, 0.52)	0.40 (0.32, 0.53)	0.40 (0.32, 0.51)	0.886
NEUT%	63.48 ± 10.22	61.02 ± 10.27	65.93 ± 9.64	0.004
LYM%	26.48 ± 9.16	28.93 ± 9.07	24.03 ± 8.64	0.001
EOS%	1.95 (1.00, 3.20)	2.30 (1.20, 3.40)	1.70 (0.90, 2.85)	0.104
BASO%	0.30 (0.20, 0.50)	0.30 (0.20, 0.50)	0.30 (0.20, 0.50)	0.459
MONO%	6.70 (5.70, 7.70)	6.40 (5.60, 7.65)	6.90 (5.95, 7.70)	0.255
RBC	4.63 (4.32, 4.93)	4.68 (4.33, 5.03)	4.58 (4.31, 4.79)	0.198
HGB	141.00 (132.25, 152.00)	142.00 (134.00, 156.50)	140.00 (132.00, 148.00)	0.17
HCT	0.43 (0.40, 0.45)	0.43 (0.40, 0.46)	0.43 (0.40, 0.44)	0.159
MCV	91.70 (88.93, 95.35)	90.70 (88.55, 95.25)	91.70 (89.70, 95.35)	0.233
MCH	30.75 (29.80, 31.40)	30.70 (29.75, 31.40)	30.80 (29.90, 31.40)	0.802
MCHC	334.00 (328.00, 340.75)	334.00 (329.00, 341.00)	334.00 (327.50, 339.50)	0.491
RDW_CV	12.70 (12.20, 13.38)	12.80 (12.30, 13.40)	12.70 (12.20, 13.25)	0.412
RDW_SD	41.60 (40.00, 43.38)	42.00 (40.10, 43.30)	41.40 (39.90, 43.90)	0.685
PLT	245.00 (202.25, 267.75)	245.00 (208.50, 262.00)	245.00 (200.50, 273.00)	0.958
MPV	10.15 (9.60, 10.90)	10.10 (9.70, 10.80)	10.20 (9.45, 10.90)	0.846
PCT	0.25 (0.22, 0.27)	0.25 (0.22, 0.27)	0.25 (0.21, 0.28)	0.651
PDW	15.90 (13.03, 16.20)	15.80 (12.35, 16.20)	15.90 (14.05, 16.25)	0.148
P_LCR	27.10 (22.30, 32.40)	27.00 (22.20, 31.05)	27.80 (23.55, 32.50)	0.727
RET	0.05 (0.04, 0.06)	0.05 (0.04, 0.06)	0.05 (0.04, 0.07)	0.917
RETP	1.05 (0.82, 1.38)	1.05 (0.85, 1.32)	1.05 (0.81, 1.42)	0.834
ALT	16.00 (13.00, 24.75)	17.00 (13.00, 26.00)	16.00 (13.00, 21.00)	0.42
AST	20.00 (16.00, 23.75)	20.00 (17.00, 24.00)	20.00 (15.50, 22.00)	0.221
ALP	72.00 (58.25, 87.00)	69.00 (58.50, 85.50)	72.00 (58.50, 87.50)	0.465
GGT	23.00 (17.00, 35.00)	22.00 (16.50, 34.00)	23.00 (18.50, 35.50)	0.548
ChE	7807.50 (7012.50, 9005.25)	8256.00 (7179.50, 9434.50)	7513.00 (6931.50, 8192.50)	0.054
TBIL	8.60 (6.55, 11.70)	9.30 (7.00, 12.20)	8.30 (6.00, 10.30)	0.058
DBIL	3.00 (2.40, 4.40)	3.40 (2.40, 4.45)	3.00 (2.35, 3.95)	0.303
IBIL	5.40 (4.10, 7.57)	6.10 (4.50, 8.35)	5.30 (3.35, 6.70)	0.016
TBA	3.50 (2.30, 5.77)	3.20 (2.05, 5.80)	3.50 (2.75, 5.35)	0.251
PA	240.00 (212.25, 280.00)	250.00 (218.00, 285.00)	240.00 (200.00, 275.00)	0.144
TP	70.30 (67.03, 74.45)	71.50 (69.20, 75.00)	69.20 (65.55, 73.50)	0.004
GLOB	29.20 (26.33, 32.68)	29.50 (26.55, 32.30)	29.10 (25.60, 33.15)	0.441
A/G	1.40 (1.20, 1.60)	1.40 (1.30, 1.60)	1.40 (1.20, 1.50)	0.111
RBP	41.05 (30.07, 50.00)	40.00 (28.55, 51.00)	41.05 (32.00, 50.00)	0.59
GLU	4.94 (4.42, 5.42)	4.88 (4.39, 5.42)	4.95 (4.62, 5.42)	0.648
UA	278.00 (237.00, 324.00)	295.00 (244.50, 337.00)	269.00 (233.50, 297.50)	0.034
Cr	66.00 (59.00, 73.00)	67.00 (60.00, 75.50)	66.00 (59.00, 71.50)	0.49
BUN	4.80 (4.00, 5.87)	4.57 (4.00, 5.95)	4.80 (4.10, 5.75)	0.695
K	3.80 ± 0.39	3.76 ± 0.37	3.85 ± 0.40	0.163
Na	141.00 (140.00, 143.00)	141.00 (140.00, 143.00)	141.00 (140.00, 143.00)	0.681
CL	102.00 (101.00, 104.00)	103.00 (101.00, 104.00)	102.00 (101.00, 103.00)	0.071
Ca	2.25 ± 0.12	2.28 ± 0.13	2.22 ± 0.11	0.005
iCa	1.19 (1.13, 1.24)	1.20 (1.13, 1.26)	1.17 (1.13, 1.22)	0.064
P	1.07 (0.93, 1.20)	1.09 (0.95, 1.19)	1.05 (0.93, 1.19)	0.713
HCO3	24.95 (23.20, 27.08)	24.40 (23.00, 26.95)	25.00 (24.00, 27.10)	0.219
AG	14.18 (12.43, 15.80)	14.10 (11.80, 15.75)	14.20 (13.15, 15.80)	0.493
OP	281.90 (278.68, 284.78)	281.90 (279.25, 284.00)	281.40 (277.90, 285.75)	0.848
TC	4.21 ± 0.83	4.35 ± 0.95	4.06 ± 0.68	0.039
TG	1.24 (0.96, 1.61)	1.24 (0.96, 1.60)	1.24 (0.96, 1.64)	0.604
HDL	1.04 (0.88, 1.27)	1.02 (0.88, 1.26)	1.04 (0.89, 1.27)	0.744
LDL	2.64 (2.19, 3.10)	2.78 (2.22, 3.26)	2.62 (2.18, 2.84)	0.076
Apoa	1.14 (1.00, 1.23)	1.14 (0.98, 1.26)	1.14 (1.04, 1.21)	0.653
Apob	0.80 (0.68, 0.86)	0.80 (0.71, 0.89)	0.80 (0.68, 0.80)	0.205
LPa	175.00 (95.75, 262.25)	175.00 (83.00, 276.00)	175.00 (106.00, 240.50)	0.876
Cys_c	0.90 (0.85, 1.00)	0.90 (0.80, 0.99)	0.90 (0.88, 1.00)	0.614
CRP	2.50 (1.00, 6.87)	2.34 (0.79, 5.73)	2.70 (1.43, 7.28)	0.17
SOD	175.00 (158.25, 181.75)	175.00 (160.50, 186.00)	175.00 (156.00, 177.50)	0.18
PNI	48.83 (46.65, 51.82)	51.90 (48.00, 55.20)	47.75 (45.15, 49.38)	<0.001
Sex	32 (22.54%)	17 (23.94%)	15 (21.13%)	0.841
	110 (77.46%)	54 (76.06%)	56 (78.87%)	

## 4 Discussion

In this study, 192 patients with NPC were included, among whom 60.94% developed grade 2 or higher RIOM, indicating that moderate-to-severe RIOM remains a frequent toxicity during radiotherapy for NPC. Through integrated feature selection using LASSO, RF, and SVM-RFE, followed by univariate and multivariate logistic regression analysis, the PNI was identified as the only independent factor significantly associated with grade 2 or higher RIOM. Continuous-variable analysis demonstrated that higher PNI levels were associated with a reduced risk of RIOM (OR = 0.586, 95% CI: 0.463–0.741, P<0.001). Furthermore, Firth penalized likelihood regression confirmed that patients with high PNI had a substantially lower risk of developing grade 2 or higher RIOM compared with those with low PNI (OR = 0.067, 95% CI: 0.022–0.178, P<0.001).These findings suggest that a reduced PNI is not merely an abnormal laboratory parameter but may represent an important high-risk marker for moderate-to-severe RIOM in patients with NPC receiving chemoradiotherapy.

The PNI-based nomogram achieved AUC values of 0.842 and 0.725 in the training and internal validation cohorts, respectively, demonstrating strong discrimination in the training cohort and acceptable predictive performance in the validation cohort. The calibration curve showed good agreement between predicted probabilities and observed outcomes, while DCA suggested potential clinical utility within a specific range of threshold probabilities. The decreased AUC in the internal validation cohort compared with the training cohort indicates a reduction in predictive performance when applied to new samples, which may be attributable to the limited sample size and potential optimism bias during model development. Therefore, the current model should be regarded as a preliminary risk stratification tool rather than a substitute for comprehensive clinical assessment. In sensitivity analysis using PSM, the association between PNI and grade 2 or higher RIOM remained statistically significant (OR = 0.709, 95% CI: 0.627–0.800, P<0.001), and the matched cohort model achieved an AUC of 0.810, further supporting the robustness of the primary findings.

Our findings are consistent with the previous study by Liu et al., which demonstrated that dynamic changes in PNI during radiotherapy were associated with the risk of severe oral mucositis in patients with head and neck cancer (18). PNI integrates serum albumin concentration and peripheral blood lymphocyte count, thereby reflecting both nutritional reserves and systemic immune status. Radiation exposure can induce excessive production of reactive oxygen species (ROS), triggering oxidative stress, DNA damage, inflammatory mediator release, and apoptosis of mucosal epithelial cells, ultimately leading to disruption of the oral mucosal barrier (19). Meanwhile, radiation-induced lymphopenia may impair immune regulation and tissue repair capacity (20). Given the high radiosensitivity of lymphocytes, persistent lymphocyte depletion during radiotherapy may further compromise host tolerance and recovery from radiation-induced injury (21). In addition, reduced serum albumin levels generally indicate inadequate nutritional reserves and may be accompanied by impaired antioxidant defense and tissue regeneration capacity. Collectively, a low PNI may represent a composite state characterized by malnutrition, immune dysfunction, and impaired mucosal repair capacity, thereby increasing susceptibility to grade 2 or higher RIOM.

Overall, our findings support the potential application of pretreatment PNI as an early risk stratification marker for grade 2 or higher RIOM in patients with NPC. Although the PNI-based nomogram demonstrated acceptable discrimination, calibration, and potential clinical benefit, its clinical applicability requires further confirmation through independent external validation.

Several limitations of this study should be acknowledged. First, this was a retrospective, single-center study with a relatively limited sample size, which may restrict the generalizability of the findings. Second, detailed treatment-related information, including chemotherapy regimens, agents, doses, and treatment cycles, as well as radiotherapy field characteristics and dose-volume parameters, was not systematically incorporated into the present analysis. These treatment-related factors may influence the risk and severity of RIOM and therefore represent potential unmeasured confounders. Third, smoking and alcohol consumption, oral hygiene status, preventive dental interventions before radiotherapy, and previous or concurrent antibiotic use were not systematically documented in the retrospective medical records and therefore could not be reliably included in the analysis. These factors may also contribute to residual confounding. Future prospective, multicenter studies with larger sample sizes should systematically collect these clinical and treatment-related variables and perform external validation to further evaluate and improve the generalizability and clinical utility of the proposed model.

## 5 Conclusion

This study established and internally validated a PNI-based prediction model for early risk stratification of grade 2 or higher RIOM in patients with NPC receiving chemoradiotherapy. Higher pretreatment PNI levels were independently associated with a reduced risk of grade 2 or higher RIOM, suggesting that PNI may serve as a readily available marker for identifying patients at elevated risk. The PNI-based nomogram provides an individualized approach to risk estimation and may facilitate pretreatment risk assessment and preventive management.

## Data Availability

The data analyzed in this study is subject to the following licenses/restrictions: Dataset access is restricted to protect patient privacy under hospital ethical management rules. The original raw data is not publicly available. Requests to access these datasets should be directed to Haoxuan Zhang.
